# Detection of DIAG and LINE Patterns in PassPoints Graphical Passwords Based on the Maximum Angles of Their Delaunay Triangles

**DOI:** 10.3390/s22051987

**Published:** 2022-03-03

**Authors:** Lisset Suárez-Plasencia, Joaquín Alberto Herrera-Macías, Carlos Miguel Legón-Pérez, Guillermo Sosa-Gómez, Omar Rojas

**Affiliations:** 1 Instituto de Criptografía, Facultad de Matemática y Computación, Universidad de la Habana, Habana 10400, Cuba; lisset.suarez@matcom.uh.cu (L.S.-P.); joaquin.herrera@matcom.uh.cu (J.A.H.-M.); clegon58@gmail.com (C.M.L.-P.); 2 Facultad de Ciencias Económicas y Empresariales, Universidad Panamericana, Álvaro del Portillo 49, Zapopan 45010, Jalisco, Mexico; gsosag@up.edu.mx; 3 Faculty of Economics and Business, Universitas Airlangga, Surabaya 60286, East Java, Indonesia

**Keywords:** PassPoints, graphical passwords, DIAG patterns, LINE patterns, maximum angles of a Delaunay triangle

## Abstract

An alternative authentication method to traditional alphanumeric passwords is graphical password authentication, also known as graphical authentication, for which one of the most valuable cued-recall techniques is PassPoints. This technique stands out for its security and usability. However, it can be violated if the user follows a predefined pattern when selecting the five points in an image as their passwords, such as the DIAG and LINE patterns. Dictionary attacks can be built using these two patterns to compromise graphical passwords. So far, no reports have been found in the state of the art about any test capable of detecting graphical passwords with DIAG or LINE patterns in PassPoints. Studies carried out in other scenarios have shown the effectiveness of the characteristics of Delaunay triangulations in extracting information about the dependence between the points. In this work, graphical passwords formed by five randomly selected points on an image are compared with passwords whose points contain patterns of the DIAG or LINE type. The comparison is based on building for each password its Delaunay triangulation and calculating the mean value of the maximum angles of the triangles obtained; such a mean value is denoted by *amadt*. It is experimentally shown that in passwords containing DIAG and LINE patterns, the value of *amadt* is higher than the one obtained in passwords formed by random dots. From this result, it is proposed to use this *amadt* value as a statistic to build a test of means. This result constitutes the work’s main contribution: The proposal of a spatial randomness test to detect weak graphic passwords that contain DIAG and LINE type patterns. The importance and novelty of this result become evident when two aspects are taken into account: First, these weak passwords can be exploited by attackers to improve the effectiveness of their attacks; second, there are no prior criteria to detect this type of weak password. The practical application of said test contributes to increasing PassPoints security without substantially affecting its efficiency.

## 1. Introduction

With the rapid development and widespread use of new technologies, users have more and more information that must be protected, so it becomes necessary to use secure passwords that are also easy to remember. Although there are some alternatives, the most popular authentication method and the standard on most devices and websites is still alphanumeric. Studies carried out [1,2,3,4] show that users tend to select phrases that are easy to remember or related to personal information as passwords, and thus an attacker could easily compromise them through various types of attacks. To avoid this, it is common to require a certain length of the password and the use of special characters. Although this tends to increase the security of the password, such measures greatly hinder its memorability. That is why graphic authentication systems emerge as an alternative, supported by the fact that humans more easily remember images or parts of them instead of texts [5]. Graphic passwords are an active field of current research, as evidenced by recent publications within the last two years [2,6,7,8,9,10,11].

Graphical authentication systems cover a vast number of techniques. A general description of graphical password authentication can be found in [12], while Ref. [13] discusses its security and usability. Among them, graphical authentication systems of the cued-recall type are based on the recognition of key points in images; in particular, this work focuses on one of the most advantageous techniques of this type in terms of security and usability, PassPoints [13,14], which bases its operation on the user-selection of a set of five points in an image as their password in the registration phase. There are extensive studies that show that users usually select their password’s five points following some predefined pattern, which might occur independently of the selected background image [15]. Some of the patterns reported in the literature [15,16,17,18,19,20,21] are: the patterns with a predetermined shape (Z, W, C, V), the clustered ones, the regular ones, and the LOD and DIAG patterns (or diagonal patterns) in which LINE (or line-shaped) patterns are included. This constitutes a weakness of graphical passwords since such users’ tendency to create patterns between the points that make up their passwords can be used on its own, or in conjunction with image Hotspots, by attackers to create effective dictionary attacks [16,17,18]. That is why there is a need to have tests that detect a priori the existence of these patterns in the passwords established by users, as they would help to increase the security of the PassPoints technique significantly.

Few reports have been found on research directed on this aspect in recent years. In [9,10], it was experimentally demonstrated that some of the most used classical tests in Complete Spatial Randomness like the K-Ripley function test, the *G* function test or the distance to the nearest neighbor test, and the *F* function or empty space distance are not very effective at detecting graphical passwords made up of clustered or regular patterns in PassPoints scenario. Consequently, in [10] a test that allows for effective detection of graphical passwords formed by clustered and regular patterns in PassPoints, based on the average distance between points, was proposed. So far, no reports have been found in state of the art about any test capable of detecting graphical passwords with DIAG or LINE patterns in PassPoints.

Considering that the characteristics of a Delaunay triangulation allow for the extraction of information about the dependence between points, they have been used as a tool in other scenarios since the mid-1980s to detect patterns of points. In [22], Chiu used several of these characteristics to detect clustering and regularity between points. In particular, the characteristic of the “maximum angle of a Delaunay triangle”, in the bibliography consulted, was never used to detect other types of patterns in addition to the clustered or regular ones. However, given that the DIAG and LINE patterns are characterized by having an angle close to $0^{\circ }$ between two consecutive segments [15], in this work, the hypothesis is proposed: The mean of the maximum angles of the Delaunay triangles formed from the points of the graphic passwords of PassPoints, is an effective statistic to detect the existence of DIAG or LINE patterns in those passwords, despite the small number of points. In [15], it was understood as the angle formed between two consecutive segments, the smaller of the two angles that form the intersection of the prolongation of the two segments, where a segment is formed by the union of two consecutive points of a password. We will refer to the larger of these two angles as the adjacent angle between two segments.

This work evaluates the effectiveness of the “maximum angle of a Delaunay triangle” feature to detect DIAG and LINE patterns in PassPoints passwords. For this, a statistical test is proposed, based on the average of the maximum angles of its Delaunay triangles, to detect these passwords. The effectiveness of the proposed test is verified experimentally by estimating the probability of type I and type II errors. The test is designed to be included in graphical authentication systems of the cued-recall type, similar to PassPoints, allowing a user to be warned during the registration phase about the selection of a possible password with a DIAG or LINE pattern, or the password could even be rejected, thus increasing the security of the system. All the experiments were carried out taking as a reference a resolution image of 1920×1080 pixels, as it is one of the most used digital devices and complies with the 16:9 ratio, considering the current standard. In addition, the experiments were run in the R2018a version of Matlab, using a Laptop PC with an Intel (R) Pentium processor (R) CPU N5000 @ 1.10 GHz (2 CPUs), ∼1.6 GHz, and 4 GB of RAM. In Matlab versions up to the R2006b, bugs were found in its random number generator [23]. Instead, Matlab, in its R2018a version has the pseudorandom number generator, *Threefry* [24]. In [24], it is shown that *Threefry* satisfies two important conditions: first, it is a fast pseudorandom number generator; second, the randomness of its output was checked by applying a battery of randomness tests. This aspect is of vital importance in this investigation since our point of reference is the graphic passwords whose points follow a random pattern.

The remainder of this work is structured into four sections: Section 2 consists of a brief description of PassPoints, DIAG and LINE patterns, and Delaunay triangulation. Section 3 shows our main contribution: the detection of DIAG and LINE patterns in PassPoints graphical passwords. Related works are presented in Section 4. Finally, Section 5 presents the conclusions and possible future lines of research.

## 2. Preliminaries

### 2.1. PassPoints

PassPoints is a cued-recall type technique designed in 2005 by Wiedenbeck et al. [14]. A user must select an ordered set of five points (pixels) as his password in the registration phase in an image provided by him or the system. The user will have to select approximately the same points chosen in their registration phase and the same order in the authentication phase. However, not all images are suitable for PassPoints, since one of the ways to attack this technique is through the so-called Hotspots, which are nothing more than the points most likely to be selected in an image, which is why in [12] it is recommended that, for greater security of this technique, the user or system selects an image with hundreds of Hotspots scattered from an homogeneous shape. In [3], they proposed a method that allows evaluating whether an image is suitable to be used in this technique. Such a method derives from a model developed to identify in an image the most probable regions that users usually select when forming their password. The said model can predict Hotspots with effectiveness between 70% and 80% according to its experiments; however, the sample size of their experiments is small. Using the proposed model in PassPoints would be very useful to increase reliability in image allocation. On the other hand, in [4], they showed how a small change in the image affects the password selected by the user in the registration phase and, therefore, its security.

In [25], a study was made of various current methods of graphical authentication, PassPoints standing out among them for its security and usability. However, [13] highlights the influence of the human factor in choosing non-random passwords in PassPoints. To facilitate the memorability of their passwords, users tend to select points that follow different patterns [15] independent mainly of the background image. These patterns are predictable and can be exploited by an attacker to obtain the password, so graphical passwords must follow a random pattern to maintain their security. Another weakness in the operation of this technique lies in the fact that the user may take time to select the points that make up their password in the registration or authentication phase, causing possible attacks of the Shoulder-Surfing type [13].

### 2.2. DIAG and LINE Patterns

**Figure 1:**
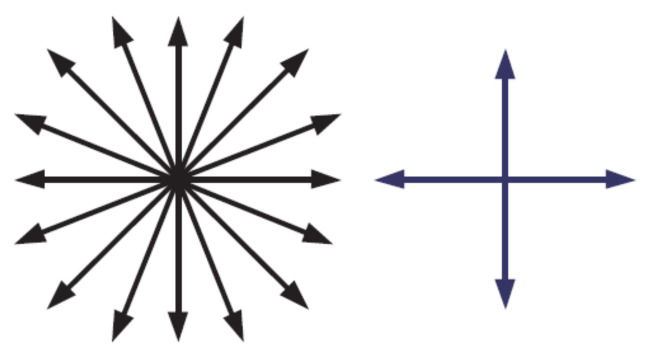
DIAG (**left**) and LINE (**right**) patterns. Source:Recovered from Van Oorschot, P. C., Salehi-Abari, A. & Thorpe, J. (2010). Purely Automated Attacks on PassPoints-Style Graphical Passwords. IEEE Transactions on Information Forensics and Security.

Among the non-random patterns that users usually select as their password in the PassPoints registration phase are the DIAG and LINE patterns, see Figure 1. In [16,17,18,19,20,21], DIAG patterns were characterized as points that are found forming arcs in both horizontal and vertical directions; in contrast, the LINE patterns in [17] are characterized by being horizontal and vertical lines and are defined as a subset of the DIAG patterns.

These patterns have been effectively used to attack and obtain passwords in PassPoints systems. These attacks are based on trying all predefined words or phrases, called entry, on guessing a password or key. In the case of PassPoints, the dictionary entries correspond to sets of five points that determine a graphic password. In [16], DIAG patterns were used to build a dictionary with approximately 11 bits less than those built for Human-Seeded attacks, but that reports similar effectiveness to the latter. On the other hand, in [17,18], after carrying out a study of 223 graphical passwords in the scenario of PassPoints selected by students in two different images with an affordable number of Hotspots evenly distributed in each one of them, the authors managed to obtain from 48.2% to 54.1% of them using a $2^{35.26}$ entry dictionary attack, built using DIAG patterns and from 23.7% to 52.3% of those passwords using a $2^{29.02}$ inputs using LINE patterns.

### 2.3. Delaunay Triangulation

A Delaunay triangulation is known as the dual of a two-dimensional Voronoi diagram. Although this geometric structure was exposed before 1872 by the French mathematician Charles Delaunay [26], in 1934, the Russian mathematician Boris Nikolaevich Delone was the one who defined it. Delone himself used the French form of his last name, Delaunay, in appreciation of his French ancestors. A Delaunay triangulation is constructed given an initial set $P=\{p_{1},\ldots ,p_{n}\}$ of *n* points in the plane. These points are usually called sites, centers, or generators. A Delaunay triangulation is defined on a set *P* of points on the plane, if and only if the circumscribed circumference of any triangle in the network does not contain a point *P* in its interior. This condition is known as Delaunay’s condition [27,28,29].

A Delaunay triangulation has the following elementary properties:

- The outer boundary of the Delaunay triangulation forms the convex envelope of the set of points.
- The minimum angle within all the triangles is maximized; results with too sharp angles are avoided. As a consequence of the above, the triangles generated in a Delaunay triangulation tend to be as equilateral as possible. This is because every non-equilateral triangle always has some angle less than $60^{\circ }$.
- Triangulation is unique when no boundary edge contains more than three vertices of the lattice.

They also have the following characteristics: area and perimeter of a Delaunay triangle, the radius of a circumscribed circle in a Delaunay triangle, interior angle of a Delaunay triangle, minimum angle, mean angle, and maximum angle of a Delaunay triangle [11]. An important and interesting result to analyze if the construction of a Delaunay triangulation is correct or not is through Euler’s formula since given a set *P* of *n* sites if an amount *h* of them are found in the convex hull is obtained by a said formula that the Delaunay triangulation has 2n−2−h triangles and 3n−3−h edges [30].

There are several algorithms for the construction of a Delaunay triangulation. According to the different construction processes, the Delaunay triangulation generation algorithms can be clustered into three categories: the growth of the triangulation, the divide and conquer algorithm, and the point-by-point insertion method. Among the main algorithms for the construction of a Delaunay triangulation are: the brute force algorithm [27], the edge rotation algorithm [31], the Bowyer–Watson incremental algorithm [32,33], the sweep line algorithm (or Fortune algorithm) [34], and the recursive divide and conquer algorithm [35]. A summary of the comparison of some of these algorithms regarding the order of complexity is shown in Table 1 of [36]. For more information on the comparison between the algorithms, see [37,38].

The algorithm used in this work to construct the Delaunay triangulation was the Fortune algorithm since this algorithm is implemented in the R2018a version of Matlab and has a complexity order of O(nlogn). However, since only five points are available, great features of the laptop are not needed. In this study’s two-dimensional case of interest, the Delaunay triangulation in this software is obtained using the “delaunay” function, which creates a two-dimensional Delaunay triangulation from the points in the vectors *x* and *y*. Although it can also be calculated through the function “delaunayn”, which is used to calculate this geometric structure of the function “convulln”, which is based on the Quickhull algorithm for convex envelope (called by Qhull) proposed in [39]. For better information on the practical application of the Qhull algorithm, see [40]. There are other software and programming languages in which Delaunay triangulation is also implemented, such as R, C++, Python, and Geogebra.

## 3. Our Main Contribution: Detection of DIAG and LINE Patterns in PassPoints Graphical Passwords

### 3.1. Rationale of the Proposed Statistic

This work demonstrates that the characteristic “maximum angle of a Delaunay triangle” can be used to detect graphical passwords that follow DIAG and LINE patterns. Our hypothesis is based on the characteristic shape of these patterns. In this sense, Ref. [15] shows that, in PassPoints, the patterns that users mostly select in their passwords tend to create an almost straight line between their points. According to their study, the most common angles formed between two consecutive segments are between $0^{\circ }$ and $45^{\circ }$. This characterization is equivalent to affirming that the angle adjacent to the one formed by the two segments, and therefore the maximum angle of the triangle that they determine will have a minimum amplitude of $135^{\circ }$. This suggests that the maximum angle of each triangle will give information about the linearity between the points that form the password since at least we would have three triangles formed by the five points of the password, the “average of the maximum angles of the Delaunay triangulation” (denoted by the acronym *amadt*) is taken as a statistic to evaluate our hypothesis. To verify our hypothesis, the following experiment was designed:

**Experiment # 1: Estimation and comparison between the mean of random graphical passwords and with DIAG and LINE patterns.**

**Design of Experiment # 1**

In order to compare the mean of the *amadt* between passwords that follow a DIAG or LINE pattern and those that do not, three databases were generated by the authors for this study in an image with dimensions of 1920×1080:

- Db.Random: made up of 1000 *amadt* of randomly generated graphical passwords.
- Db.DIAG: made up of 1000 *amadt* of graphical passwords that follow a DIAG pattern, these passwords were generated by setting the angle between two consecutive segments in the interval $\left[0^{\circ },45^{\circ }\right]$.
- Db.LINE: made up of 1000 *amadt* of graphical passwords that follow a LINE pattern, these passwords were generated by setting the angle between two consecutive segments in the interval $\left[0^{\circ },45^{\circ }\right]$.

The mean was found for each of these databases, and their frequency histogram was plotted for comparison.

**Results of Experiment # 1**

**Figure 2:**
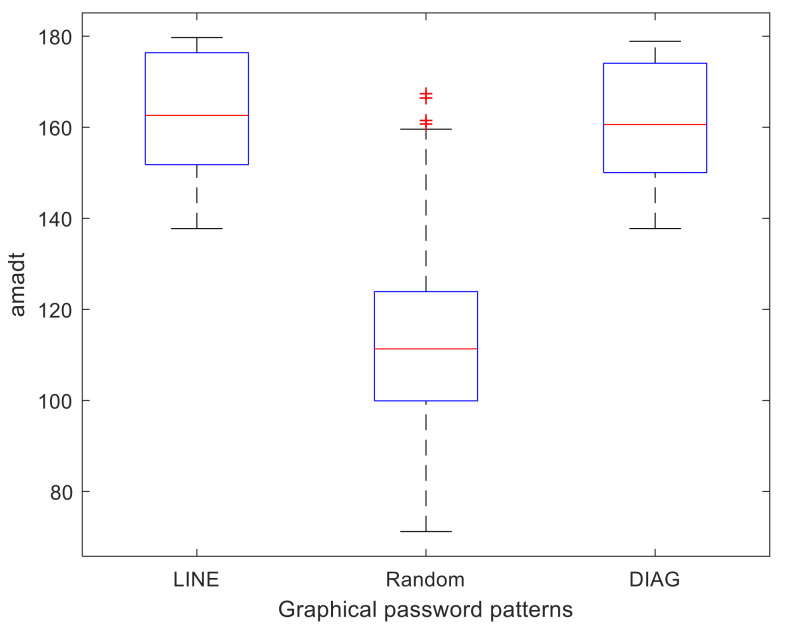
Box plot of the Db.LINE, Db.Random, and Db.DIAG.

**Figure 3:**
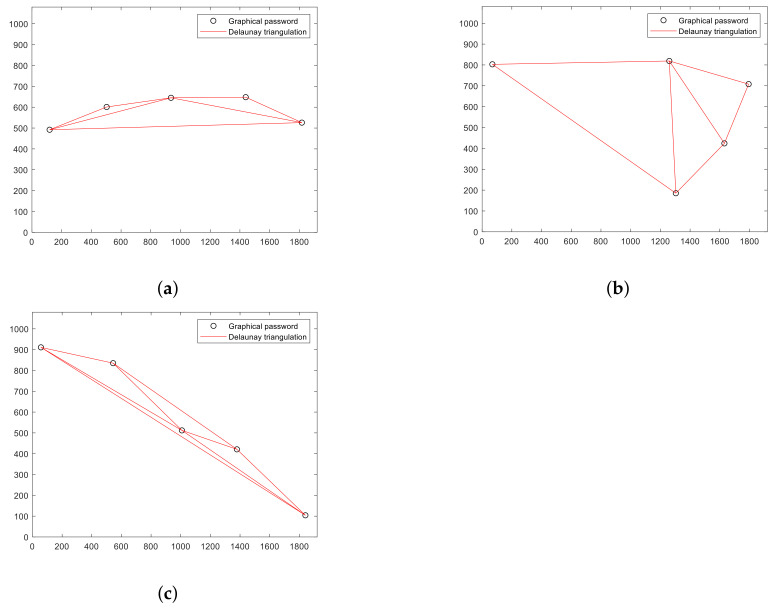
Delaunay triangulation of a graphical password with LINE (**a**), random (**b**), and DIAG (**c**) pattern. Their *amadt* are $161^{\circ }$, $83^{\circ }$, and $162^{\circ }$, respectively.

**Figure 4:**
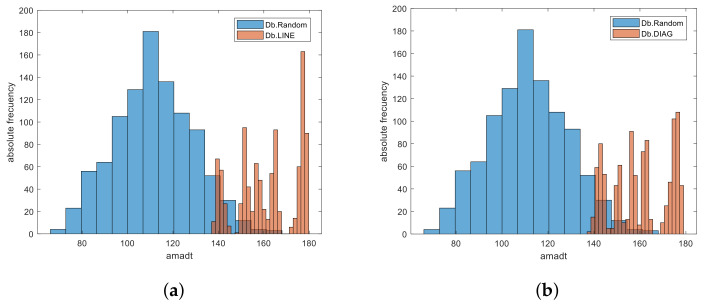
Frequency histograms of the Db.Random vs. Db.LINE (**a**) and Db.Random vs. Db.DIAG (**b**).

Figure 2 shows the box plot of each of these databases, and it can be seen how the estimated mean of the databases with DIAG (Db.DIAG) and LINE (Db.LINE) patterns is significantly higher than that estimated for the random ones (Db.Random). Figure 3 illustrates the *amadt* of three of the generated graphical passwords, one random and the other two with LINE and DIAG patterns, respectively; the almost linear shape of these last two passwords guarantees a high statistic value compared to the random password. The comparison between the frequency histograms of the Db.Random with each of the Db.LINE and Db.DIAG respectively are reflected in Figure 4. It is observed as the *amadt* of the passwords that follow these patterns shift towards the right tail of the estimated distribution for the *amadt* of the random passwords as the width of the angle between the consecutive segments of the graphical password points decreases.

**Discussion of the Results of Experiment # 1**

The results obtained support the hypothesis formulated that the mean statistic of the *N* maximum angles of the *N* Delaunay triangles of a graphical password effectively discerning between graphical passwords with DIAG or LINE patterns and random graphical passwords. This result suggests the creation of a test that uses the *amadt* as a statistic to evaluate the null hypothesis that a PassPoints graphical password does not follow a DIAG or LINE pattern against the alternative that it does. It is then necessary to determine the probability distribution that best fits the estimated distribution of the average of the maximum angles of the Delaunay triangles of random graphical passwords in PassPoints. For this purpose, the following experiment is carried out.

**Experiment # 2: Estimation of the Distribution of the Average of the Maximum Angles of the Delaunay Triangulation.**

**Design of Experiment # 2**

To estimate the distribution of the average of the maximum angles of the Delaunay triangulation triangles of the five points of a graphical password in PassPoints, 1000 graphical passwords of five points were randomly generated in an image of dimensions 1920×1080. A corresponding Delaunay triangulation was constructed for each of these passwords, and the average of the maximum angles of the triangles resulting from their Delaunay triangulation was determined. The database containing the 1000 *amadt* was named DB.1.1. To measure the fit of the *amadt* to some known theoretical distribution, Matlab 2018 software was used, from which it was possible to obtain the following results.

**Experiment results #2**

**Figure 5:**
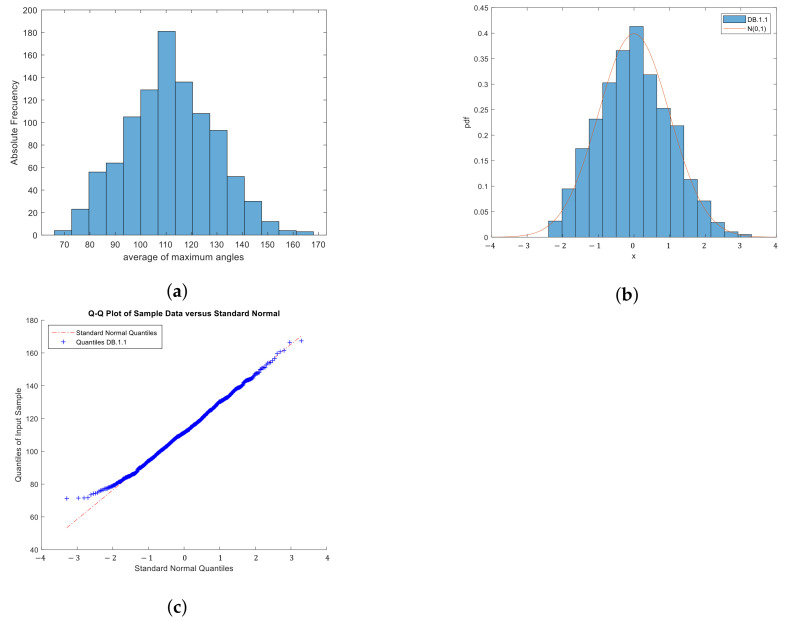
Histogram of frequencies of the *amadt* of each password of DB.1.1 (**a**) and its adjustment to a standard Normal N(0,1) (**b**), Q-Q plot (**c**).

The results obtained are reflected in Figure 5 and Table 1 and Table 2.

**Discussion of the results of Experiment # 2**

Figure 5 shows the frequency histogram of the *amadt* contained in DB.1.1. The *amadt* seem to come from a normal distribution, so the fit to this distribution was measured. It was estimated that the intervals cover the parameters μ∈[110.8,112.9] and σ∈[16.5,18] with 95% probability, the mean value of each interval was taken as a point estimate of the parameters μ=111.8 and σ=17.2. These parameter estimates and the estimated normal distribution are summarized in Table 1. To measure the adjustment of the sample to a standard normal distribution, three normality tests were applied: Anderson–Darling, Kolmogorov–Smirnov, and Chi-Square. In all three cases, the adjustment to a normal distribution with α=0.05 is accepted, as shown in the Table 2.

Figure 5b,c show the visual adjustment of the *amadt* contained in DB.1.1 to a standardized normal. Based on these results, it is assumed in the rest of the work that the sample DB.1.1 and, therefore, the average of the maximum angles of the Delaunay triangulation of a set of five random points come from a Normal distribution. The importance of this result lies in the fact that the Normal distribution of *amadt*, despite being a mean, is not a direct consequence of the Central Limit Theorem. Given that the said theorem argues the Normal distribution of the mean for large values of the sample size *N*, in practice, it is usually assumed for N>30 [41]; however, in the proposed case, it was obtained experimentally that 3≤N≤5.

### 3.2. Proposed Test for the Detection of Passwords Formed by DIAG or LINE Patterns in PassPoints

Since the *amadt* distributes Normal, this subsection proposes a one-tailed (right) test for the mean of a Normal distribution to detect graphical passwords that follow a DIAG or LINE pattern in the PassPoints scenario. Those points whose *amadt* is significantly higher than the mean can be considered to follow a DIAG or LINE pattern; on the contrary, those with an *amadt* close to or lower than the mean do not follow a DIAG or LINE pattern. Since this test is based on the width of the angles and not on the length of the sides or other characteristic that varies with the image size, it will be valid for all sizes of images with a 16:9 ratio. Since images with this relationship will have proportional dimensions and their corresponding triangles will be similar, the proposed test can be formalized as follows:

1. Random variable *X*: maximum angle of one of the Delaunay triangles in a set of 5 points.
  - The sample size, *N*, varies from 3 to 5 since experimentally it was found that this is the range in which the number of triangles of the Delaunay triangulation of a set of 5 points oscillates.
2. Statistician: $Z=\frac{\bar{X}-117.8}{17.2}$, $\bar{X}:amadt$
  - In the previous section it was shown that amadt distributes Normal so that the test statistic *Z* follows a distribution N(0,1) despite the small sample.
3. Null hypothesis: $H_{0}:E\left(Z\right)=0$
  - The average of the maximum angles observed in the Delaunay triangulation is equal to the expected mean under the randomness hypothesis of the five points of the password estimated in Section 3.1, so this hypothesis is equivalent to the fact that the points of the graphical password have been selected randomly.
4. Alternative hypothesis: $H_{a}:E\left(Z\right)>0$
  - The average of the maximum angles observed in the Delaunay triangulation is greater than the expected mean under the randomness hypothesis of the five points of the password estimated in the Section 3.1, so this hypothesis is compatible with the fact that the points of the graphic password follow a DIAG or LINE pattern.
5. Rejection region: $\left\{z:Z>z_{\alpha }\right\}$, where α is the significance level set by the user or system.

### 3.3. Implementation of the Proposed Test

For the implementation of the proposed test, the coordinates $\left(x_{i},y_{i}\right)$ of each of the $p_{i}$ points, i=1,…,5 are taken as input values. From these points, its Delaunay triangulation is constructed using one of the known algorithms; in the case of Matlab, it can be calculated as mentioned above using the command *delaunay*, which in turn uses the Fortune algorithm. Soon the length of the sides of each triangle will be determined by the Euclidean distance of two to two of the points that form it, denoted by $p_{r}$, $p_{j}$ and $p_{k}$.

$$a=\left|\right|p_{r},p_{j}{\left|\right|}_{2},\;b=\left|\right|p_{r},p_{k}{\left|\right|}_{2},\;c=\left|\right|p_{j},p_{k}{\left|\right|}_{2}.$$

To find the angles of each triangle, the Law of Cosines is applied:

$$\theta =arccos\left(\frac{a^{2}+b^{2}-c^{2}}{2ab}\right),\phi =arccos\left(\frac{a^{2}+c^{2}-b^{2}}{2ac}\right),\psi =arccos\left(\frac{c^{2}+b^{2}-a^{2}}{2cb}\right).$$

It remains to find the maximum of these three angles max{θ,ϕ,ψ}. It is analogous for the remaining triangles of the Delaunay triangulation. Once the maximum angles of the Delaunay triangulation have been obtained, its mean is calculated, with which the value of the *Z* statistic is found. The null hypothesis is rejected depending on whether the test statistic *Z* belongs to the rejection region for the established α. A possible implementation of this test in Matlab 2018 would be:
function [*h*]=ProposedTest(*xy*,*z*)% *xy* is a 5×2 matrix, where each row *i* represents the coordinates $\left(x_{i},y_{i}\right)$ of point *i*.% *z* is the critical value determined by the preset significance level.% The test returns *h* = 0 if the null hypothesis is rejected, *h* = 1 otherwise.*A* = [];*t* = delaunay(*xy*(:,1),*xy*(:,2));*d* = pdist(*xy*);*m* = squareform(*d*);for *i* = 1:length(*t*)        θ=$acos(\left({\left(m\left(t\left(i,1\right),t\left(i,3\right)\right)\right)}^{2}+{\left(m\left(t\left(i,2\right),t\left(i,3\right)\right)\right)}^{2}-\left(m{\left(t\left(i,1\right),t\left(i,2\right)\right)}^{2}\right)\right)$                /(2∗m(t(i,1),t(i,3))∗m(t(i,2),t(i,3))));        ϕ=$acos(\left({\left(m\left(t\left(i,1\right),t\left(i,2\right)\right)\right)}^{2}+{\left(m\left(t\left(i,2\right),t\left(i,3\right)\right)\right)}^{2}-\left(m{\left(t\left(i,1\right),t\left(i,3\right)\right)}^{2}\right)\right)$                /(2∗m(t(i,1),t(i,2))∗m(t(i,2),t(i,3))));        ψ=$acos(\left({\left(m\left(t\left(i,1\right),t\left(i,2\right)\right)\right)}^{2}+{\left(m\left(t\left(i,1\right),t\left(i,3\right)\right)\right)}^{2}-\left(m{\left(t\left(i,2\right),t\left(i,3\right)\right)}^{2}\right)\right)$                /(2∗m(t(i,1),t(i,2))∗m(t(i,1),t(i,3))));        *A* = [*A*,max([θ,ϕ,ψ])*180/pi];end*Z* = (sum(*A*)/length(*t*)-117.8)/17.2;if Z>z     *h* = 0;else     *h* = 1;end

The main complexity of this implementation of the proposed test is the use of the *delaunay* function of the Matlab software. This function, as mentioned before, makes use of the Fortune algorithm to construct the triangulation; its complexity is of the order O(nlog(n)) [34], which represents the minor complexity among the algorithms that build it. The rest of the operations are elementary operations, and their complexity is linear. Therefore this implementation does not significantly affect the efficiency of the graphical authentication system.

### 3.4. Estimate of the Probability of the Type *I* and Type II Errors Made by the Test

**Experiment #3: Estimation of the probability that the proposed test will make a type I error**

**Design of Experiment # 3**

To estimate the probability that the test decides that the graphical password contains a DIAG or LINE pattern when, in fact, it follows a random pattern (error of type *I* or false positives), a DB.1.2 database was generated with 10,000 graphic passwords randomly on the image (passwords without DIAG and LINE patterns). The test described in Section 3.3 was applied to each of these graphical passwords for the values of $z_{\alpha }\in \left\{0.842,1.282,1.645,2.054,2.326\right\}$ corresponding to the levels of significance α∈{0.2,0.1,0.05,0.02,0.01}.

**Experiment results #3**

**Figure 6:**
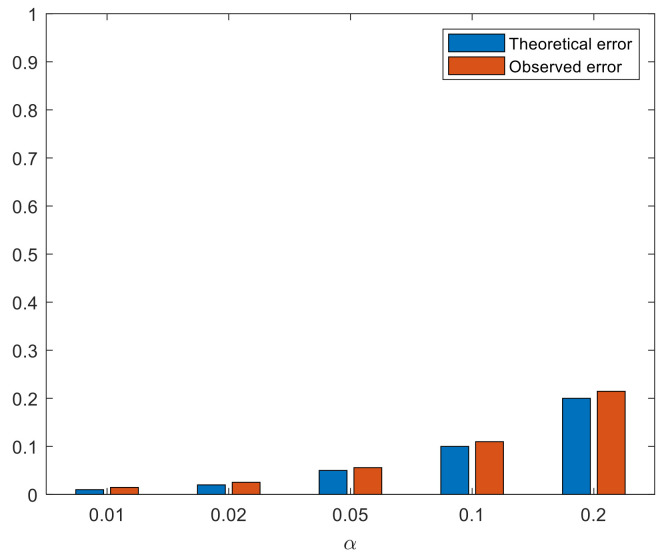
Comparison between the theoretical (α) and estimated ($\hat{\alpha }$) probabilities of committing an error of type *I*.

The results obtained are reflected in Table 3 and in Figure 6.

**Discussion of the results of Experiment # 3**

The estimated probabilities of committing an error of type *I* ($\hat{\alpha }$) by the test are adjusted to the preset theoretical probabilities (α), see Figure 6.

**Experiment #4: Estimation of the probability that the proposed test will make a type II error**

**Design of Experiment # 4**

To estimate the probability that the test detects a graphic password as random when it follows a DIAG or LINE pattern (type error II or false negative), the databases of patterns LINE (Db.LINE) and DIAG (Db.DIAG) proposed in Section 3.1 were expanded until obtaining 30,000 graphical passwords. In turn, each of these databases was subdivided into three 10,000 passwords each (Db1.Line, Db2.Line, Db3.Line) and (Db1.Diag, Db2.Diag, Db3.Diag), each with a difference of $15^{\circ }$ between two consecutive segments greater than the previous one.

- Db1.Line: graphical passwords that follow a LINE pattern with a maximum width between two consecutive segments of $15^{\circ }$.
- Db2.Line: graphical passwords that follow a LINE pattern with a maximum width between two consecutive segments that varies between $15^{\circ }$ and $30^{\circ }$.
- Db3.Line: graphical passwords that follow a LINE pattern with a maximum width between two consecutive segments that varies between $30^{\circ }$ and $45^{\circ }$.
- Db1.Diag: graphical passwords that follow a DIAG pattern with a maximum width between two consecutive segments of $15^{\circ }$.
- Db2.Diag: graphical passwords that follow a DIAG pattern with a maximum width between two consecutive segments that varies between $15^{\circ }$ and $30^{\circ }$.
- Db3.Diag: graphical passwords that follow a DIAG pattern with a maximum width between two consecutive segments that varies between $30^{\circ }$ and $45^{\circ }$.

**Experiment results #4**

The results obtained by the test for each of the databases with LINE patterns and with DIAG patterns, for the preset significance levels, are shown in Table 4 and Table 5, respectively.

**Discussion of the results of Experiment # 4**

The proposed test showed during this experiment a high effectiveness to detect graphical passwords with a DIAG or LINE pattern, reaching 100% of passwords whose amplitude between two segments varies between $0^{\circ }$ and $30^{\circ }$ for any of the experienced significance levels and also the 100% of those corresponding to the database Db3.Line and Db3.Diag for the levels α=0.2 and α=0.1. For passwords that vary between $30^{\circ }$ and $45^{\circ }$ detect around 88% for the significance level α=0.05, approximately 42% with α=0.02 and a 16% for α=0.01.

**Figure 7:**
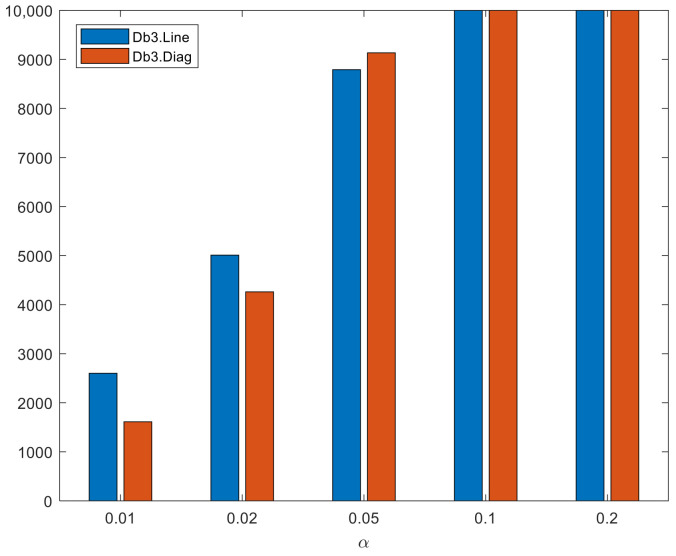
Comparison between the number of graphical passwords detected in the Db3.Diag and Db3.Line.

The levels of detection of passwords made up of DIAG and LINE patterns are quite similar. However, in general those of LINE patterns are higher, reaching for α=0.02 an 8% more than LINE patterns in passwords whose angles between two consecutive segments have an amplitude between $30^{\circ }$ and $45^{\circ }$ to each other, and an additional 10% for α=0.01, as shown in Figure 7. The similar detection values between both patterns indicate that the test does not depend greatly on the orientation of the pattern but rather depends more on the amplitude of the angles formed between two consecutive segments of the password, which determines the linear shape or not of the pattern.

### 3.5. Discussion of the Results of the Proposed Test

The Experiments #3 and #4 clearly show the effectiveness of the proposed test in detecting graphical passwords formed by DIAG and LINE patterns in the PassPoints scenario. Patterns whose angles between consecutive segments are between $0^{\circ }$ and $30^{\circ }$, which are closest to the typical shape of DIAG and LINE patterns and which have been simulated in the databases of data Db1.Diag, Db2.Diag, Db1.Line, Db2.Line are detected by the proposed test with effectiveness of 100% for all the α analyzed. On the other hand, the simulated patterns in the Db3.Diag and Db3.Line databases are the furthest from the ideal DIAG and LINE form, and their effectiveness does vary with the established α. That is why they are taken as a reference to conclude the effectiveness of the proposed test, as summarized in Table 6.

The level α=0.05 is recommended for general cases since it allows the detection of more than 88% and 91% of passwords with DIAG and LINE patterns, respectively, and only allows one false positive out of every 20 passwords. For its part, α=0.01 allows a high level of security by detecting 100% of passwords with DIAG or LINE patterns but allows one false positive out of 10. The user or system is free to select the α that best suits their needs. The results obtained show that the effectiveness of the test to detect passwords with DIAG and LINE patterns increases as the value of the password *amadt* grows, since passwords with a higher degree of linearity will be detectable more easily and with less probability of error.

It could be debated whether passwords whose consecutive segments form angles close to but greater than $45^{\circ }$ can be considered to follow a LINE or DIAG pattern. This work is not intended to establish a limit to what extent LINE or DIAG patterns can be considered or not. Passwords whose consecutive segments exceed $45^{\circ }$ are not analyzed in this work because the studies carried out in [15] clearly show that such passwords can occur under the hypothesis of a random selection of password points. Therefore they are not considered weak passwords, and it is not of interest to detect them.

## 4. Related Works

In detecting patterns in graphical passwords in PassPoints, little has been investigated. The authors in the reviewed references agree that users should avoid predictable patterns in their passwords; however, they do not provide or make references to methods capable of detecting such patterns. Only three references have been found oriented to this topic [9,10,11], all of them very current. In [9] the effectiveness of two classic spatial randomness tests, the K-Ripley test and the nearest neighbor test, in detecting clustered patterns in PassPoints graphical passwords are evaluated; their results showed the ineffectiveness of these tests because the five points that make up a graphical password constitute a very low sample. With the same purpose, in [11] the Voronoi polygons of clustered graphical passwords were built to later evaluate the effectiveness of the “number of sides” characteristic through the criteria based on entropy and the expected value; both criteria were ineffective in the detection of these passwords. Finally, in [10] a test of proven effectiveness was proposed for the detection of clustered and regular graphical passwords in PassPoints, for which they prove that the average distance between the points of a random password distributes N(0,1), a fact that they used to construct a test of means. Regarding the DIAG and LINE patterns, even though the high frequency with which users use these patterns in their passwords, and their consequent use in the construction of dictionary attacks is well documented in the bibliography, the proposal of a test for the effective detection of these patterns until the moment of its writing was not found.

## 5. Conclusions and Future Work

This work proposes a novel test to detect graphic passwords in PassPoints that follow a DIAG or LINE pattern, valid for all sizes of images selected by the user or system with a 16:9 ratio. The proposed test was able to detect the 10,000 simulated passwords in each of the databases Db1.Diag, Db2.Diag, Db1.Line and Db2.Line at the five significance levels analyzed. Meanwhile, for the databases Db3.Diag and Db3.Line, it detected each of the 10,000 passwords for the significance levels α=0.2 and α=0.1; for the levels α∈{0.05,0.02,0.01} it detected approximately 8800, 4200, and 1600 passwords, respectively.

For the construction of the test, it was formulated and tested as an initial hypothesis that the average of the maximum angles of the triangles of the Delaunay triangulation of the five points of a PassPoints graphical password is an effective statistic to evaluate and decide whether or not the password selected by the user follows a DIAG or LINE pattern. The experiments showed that passwords with LINE and DIAG patterns had an average of the maximum angles of their Delaunay triangulation greater than the estimated mean for passwords with randomly selected points. It was experimentally verified that the selected test statistic distributes Normally. This finally allowed for the construction of a one-tailed test (right) for the mean of a Normal distribution, which evaluates the null hypothesis that the password does not follow a DIAG and LINE pattern against the alternative that it follows a DIAG or LINE pattern and the precise estimation of the probabilities of type I and II errors.

The effectiveness of the proposed test was evaluated in six databases of 10,000 passwords each, three of DIAG patterns and three of LINE patterns; these databases were generated according to the angles between two consecutive segments that Chiasson et al. [15] in her article determines as with a frequency greater than the average. The test managed to detect 100% of the analyzed graphical passwords that follow a DIAG and LINE pattern whose consecutive segments have a maximum amplitude between $0^{\circ }$ and $30^{\circ }$ (Db1.Diag, Db2.Diag, Db1.Line and Db2.Line), for the five levels of significance studied. For DIAG and LINE patterns with a maximum width between two consecutive segments of $30^{\circ }$ and $45^{\circ }$ (Db3.Diag and Db3.Line), the test detects 100% of passwords for levels α=0.2 and α=0.1; for the significance levels α∈{0.05,0.02,0.01} the effectiveness of the test was approximately 88%, 42% and 16%, respectively. The selection of the optimal α is left to the choice of the user or system according to his requirement. However, the authors recommend the significance level α=0.05 for general purposes since it provides detection values greater than 91% for DIAG patterns and 88% for LINE patterns, allowing one false positive for every 20 passwords. For users or systems that require a higher level of security, it is recommended to use α=0.01 since it detects 100% of all passwords, at the cost of committing 1 out of 10 false positives.

The importance of the test proposed in this article becomes evident when two aspects are taken into account: first, the high frequency with which users use graphical passwords that follow DIAG or LINE patterns, considered weak because they are vulnerable to dictionary attacks; second, no method capable of detecting these types of patterns in the PassPoints scenario was found at the time of writing this article. That is why the proposed test is designed to be integrated into an authentication system with the PassPoints technique, allowing the user to be warned (and even rejected) during the registration phase about a possible graphic password with a LINE or DIAG pattern, which could be obtained in consequence by an attacker through dictionary attacks, thus increasing the security and integrity of the system without significantly affecting its efficiency.

The proposed test applies to images with a 16:9 ratio; despite this being the current standard as far as image size is concerned, we intend to evaluate other image proportions in future works. Considering that the effect of using images with a ratio other than 16:9 will be to increase or decrease their width, we hypothesize that these small variations do not vary the Normal distribution of the proposed test-statistic but rather the value of its parameters. This generalization would allow the test to be applied to other aspects that are also common, such as 16:10, the standard for 15.6 inches laptops, or 13:6, common among mobile devices, thus expanding the usability of the test.

As the experiments showed, the number of Delaunay triangles in a set of five points varies from three to five depending on the position of the points. In this work, the distribution of the average of the maximum angles of the Delaunay triangulation was estimated, regardless of the number of triangles corresponding to each password. It would be interesting as future work to estimate separately the distributions of the average of the maximum angles of the Delaunay triangulation for the sets whose triangulations are three, four, and five triangles, respectively. Since the joint distribution of these sets follows a Normal distribution, we hypothesize that each of the distributions of the sets whose triangulations contain three, four, or five triangles will also Normally distribute but with different parameters. Suppose our hypothesis is fulfilled, given a graphical password entered by a user. In that case, it is possible to verify which distribution it belongs to and apply the test with the appropriate distribution. This should improve the goodness of fit and increase the accuracy of the test.

## Figures and Tables

**Table 1:** Point and interval estimation of the mean and variance of the Normal distribution, to which the *amadt* of a set of five random points is adjusted.

μ		σ		Estimated Distribution
Interval	Estimate	Interval	Estimate	
[110.8,112.9]	111.8	[16.5,18]	17.2	N(111.8,17.2)

**Table 2:** Normality test applied to the *amadt* contained in DB.1.1, with significance level α=0.05.

	Anderson-Darling	Kolmogorov-Smirnov	Chi-Squared
Result	Accepted	Accepted	Accepted
Significance level (α)	0.05	0.05	0.05
*p*-value	0.6328	0.6506	0.2288

**Table 3:** Estimate of the probability ($\hat{\alpha }$) of the type *I* error derived by the test.

DB.1.2	Probability of the Estimated Type *I* Error				
α=0.2	α=0.1	α=0.05	α=0.02	α=0.01
$\hat{\alpha }$	0.2146	0.1098	0.0558	0.0253	0.0147

**Table 4:** Estimation of the probability of committing an error of type II by the test proposed in the LINE pattern databases.

Significance Level	Estimated Probability of Type II Error		
	Db1.Line	Db2.Line	Db3.Line
α=0.2	0	0	0
α=0.1	0	0	0
α=0.05	0	0	0.12
α=0.02	0	0	0.50
α=0.01	0	0	0.74

**Table 5:** Estimation of the probability of committing a type II error by the test proposed in the DIAG pattern databases.

Significance Level	Estimated Probability of Type II Error		
	Db1.Diag	Db2.Diag	Db3.Diag
α=0.2	0	0	0
α=0.1	0	0	0
α=0.05	0	0	0.09
α=0.02	0	0	0.57
α=0.01	0	0	0.84

**Table 6:** Effectiveness of the proposed test in detecting DIAG and LINE patterns.

Significance Level	DIAG	LINE	False Positives
α=0.2	100%	100%	1 of every 5
α=0.1	100%	100%	1 of every 10
α=0.05	91%	88%	1 of every 20
α=0.02	43%	50%	1 of every 50
α=0.01	16%	26%	1 of every 100
